# Tele-Rehabilitation Interventions for Motor Symptoms in COVID-19 Patients: A Narrative Review

**DOI:** 10.3390/bioengineering10060650

**Published:** 2023-05-26

**Authors:** Serena Cerfoglio, Paolo Capodaglio, Paolo Rossi, Federica Verme, Gabriele Boldini, Viktoria Cvetkova, Graziano Ruggeri, Manuela Galli, Veronica Cimolin

**Affiliations:** 1 Department of Electronics, Information and Bioengineering, Politecnico di Milano, 20133 Milano, Italy; serena.cerfoglio@polimi.it; 2 Orthopaedic Rehabilitation Unit and Research Laboratory in Biomechanics, Rehabilitation and Ergonomics, San Giuseppe Hospital, IRCCS Istituto Auxologico Italiano, 28824 Piancavallo, Italy; p.capodaglio@auxologico.it (P.C.); f.verme@auxologico.it (F.V.); gabrieleboldini@gmail.com (G.B.); manuela.galli@polimi.it (M.G.); 3 Department of Surgical Sciences, Physical Medicine and Rehabilitation, University of Turin, 10126 Turin, Italy; 4 Clinica Hildebrand, Centro di Riabilitazione Brissago, CH-6614 Brissago, Switzerland; p.rossi@clinica-hildebrand.ch (P.R.); v.cvetkova@clinica-hildebrand.ch (V.C.); g.ruggeri@clinica-hildebrand.ch (G.R.)

**Keywords:** COVID-19, long COVID, rehabilitation, tele-rehabilitation, functional capacity, motor rehabilitation

## Abstract

The COVID-19 pandemic brought new challenges to global healthcare systems regarding the care of acute patients and the delivery of rehabilitation programs to post-acute or chronic patients. Patients who survive severe forms of COVID-19 often report incomplete healing and long-term symptoms. The need of these patients for rehabilitation has been recognized as a public health problem. In this context, the application of tele-rehabilitation has been explored to reduce the burden on healthcare systems. The purpose of this narrative review is to present an overview of the state of the art regarding the application of remote motor rehabilitation programs for paucisymptomatic acute and post-acute COVID-19 patients, with a focus on the motor aspects of tele-rehabilitation. Following an extensive search on PubMed, the Web of Science, and Scopus, specific studies have been reviewed and compared in terms of study objectives and participants, experimental protocols and methods for home-based interventions, functional assessment, and rehabilitation outcomes. Overall, this review suggests the feasibility and the effectiveness of tele-rehabilitation as a promising tool to complement face-to-face rehabilitation interventions. However, further improvements are needed to overcome the limitations and the current lack of knowledge in the field.

## 1. Introduction

Severe acute respiratory syndrome coronavirus 2 (SARS-CoV-2) is a zoonotic human coronavirus [1,2] that was firstly noted in late December 2019 in Wuhan (Hubei Province, China). After the first reported case, the virus rapidly spread worldwide, leading to the COVID-19 pandemic, which was officially declared by the World Health Organization in March 2020 [3].

The clinical severity spectrum of COVID-19 infection has been reported to be highly variable, ranging from asymptomatic and mild-to-moderate cases, to patients requiring admission to intensive care units (ICU), and mechanical ventilation due to acute respiratory distress and/or multiorgan failure [4,5,6,7,8].

Depending on the severity of the COVID-19 infection, patients may suffer several dysfunctions [9]. After discharge from acute care, COVID-19 survivors often report incomplete healing, experiencing a wide range of long-term health problems. These cases, known in the literature as “long COVID” [10], are characterized by decreased exercise capacity, impairment in respiratory function, reduced muscular strength, joint and muscle pain, chest pain, persistent cough, and rhinorrhea. In addition to the above listed motor and respiratory symptoms, former patients may experience diarrhea, smell and taste alteration, cognitive impairment, anxiety, memory loss, and sleep disturbances [11,12,13,14,15,16].

Post-COVID patients may thus undergo a wide and heterogeneous sequelae [1,11,14,17], with a persistence of over six months, preventing their return to everyday life and negatively impacting both physical and mental performance. While long-term symptoms are expected in patients recovering from severe COVID-19, a worrying number of reports have also shown the persistence of long-term health issues [18,19] in non-hospitalized patients [13,20]. Long COVID-19 has been recognized as a public health problem [11,21], for which appropriate rehabilitation interventions from the acute to the post-acute phase are required [22,23,24,25,26].

Rehabilitation plays a key role in the functional recovery of COVID-19 patients, both at the motor and respiratory level, from the early stages of the disease, customizing the interventions and their goals according to the magnitude of the symptoms in order to restore pre-infection physical capacity [11,27,28,29]. During the COVID-19 outbreak, access to traditional rehabilitation was often impossible.

The pandemic greatly increased the pressure on healthcare services, requiring a massive engagement of resources and personnel for the curing of the acute patients. Rehabilitation units were often converted into acute-care units, and outpatient rehabilitation was drastically reduced because physiatrists were diverted to acute-care duties. Fear of becoming infected via face-to-face interaction, travel restrictions, and social distancing policies also contributed to a dramatic reduction in rehabilitative treatments for chronic outpatients [8,11,30,31,32,33,34].

In this scenario, tele-rehabilitation appears to be a valuable tool to support health-care systems in managing the rehabilitation demand for chronic and long-COVID patients.

Tele-rehabilitation is an area of telemedicine representing the clinical application of consultation, preventive actions, diagnosis, and therapy using audio-visual links and components [11,35,36,37]. Remote rehabilitation was initially designed to enable patient discharge after the acute phase, thus reducing hospital stays and costs [38,39]. Such an approach is also used for the care of patients with hospital transportation issues [26].

Tele-rehabilitation can be delivered synchronously or asynchronously, allowing for a wide range of multi-disciplinary personalized interventions (e.g. strength training, breathing exercises), also mimicking virtual reality and gaming techniques [40,41,42,43,44]. It may apply to various fields, such as neural rehabilitation, physiotherapy [45,46,47,48,49], and respiratory rehabilitation [50].

In the recent years, an increasing number of studies have explored the use of technologies and digital physiotherapy practices, highlighting the potential of this approach for managing chronic outpatients [11,51,52]. Studies report the effectiveness, validity, and non-inference in the treatment of symptoms of cardiovascular [53], neurological [54], musculoskeletal [33,45,55,56,57] and respiratory disorders [58]. Indeed, tele-rehabilitation may represent a complementary tool and, when no other options are available, an alternative approach to face-to-face interventions that can meet the needs of different patients [11,59]. Since tele-rehabilitation seems to be effective and feasible in treating patients with respiratory diseases, it has been applied in managing the rehabilitation requirements of COVID-19 survivors.

Although COVID-19 primarily affects the respiratory system, survivors often report disabling motor symptoms resulting in impaired physical capacity. This may be due to muscular deconditioning [11,60,61] following prolonged immobilization [62].

Motor symptoms following acute or chronic diseases, illness, or injuries can induce specific impairments that need to be addressed in order to optimize functional recovery and reduce disability. Motor tele-rehabilitation can complement other hospital-based interventions based on the rehabilitative goals.

Motor tele-rehabilitation programs may include mobility and strength exercises, basic aerobic tasks, and daily recommendation (e.g., walking). As for strength exercises, tasks using elastic bands, light dumbbells, and body weight exercises are usually prescribed. The number of sets, repetitions, and weekly sessions is adjusted according to the patient’s health condition.

The aim of this narrative review is to present an overview of the state of the art regarding the application of motor tele-rehabilitation programs in the care of paucisymptomatic acute and post-acute COVID-19 patients, focusing on the administration modality and protocols, the functional motor assessment of the patients, and its effectiveness in contributing to the patients’ recovery.

## 2. Materials and Methods

An extensive search of the most relevant scientific literature was conducted in December 2022, focusing on studies published during the COVID-19 pandemic years (2020–2022). The electronic search was performed on PubMed, the Web of Science, and Scopus via customized queries using specific keywords and Boolean operators in the form (((POST AND COVID) OR (LONG AND COVID) OR (ACUTE AND COVID)) AND (TELEREHABILITATION AND PROGRAM)). The selection was restricted to full articles written in English, excluding reviews, perspectives, communications, and cases studies. It was decided to include studies assessing only adult patients (≥18 years) with a previous or current COVID-19 diagnosis confirmed by a reverse transcription-polymerase chain reaction (RT-PCR) test for SARS-CoV-2 [63]. As the focus of this work was motor tele-rehabilitation, studies which administered remote rehabilitation programs, without specifying or including a motor component in their protocols, were not included.

As a narrative review aimed at summarizing the current state of the art regarding motor tele-rehabilitation in COVID patients, in order to open a general debate on the topic, it was decided not to apply standards for the critical appraisal of the studies’ quality and selection, as these approaches are typical of systematic reviews.

## 3. Results

**Figure 1:**
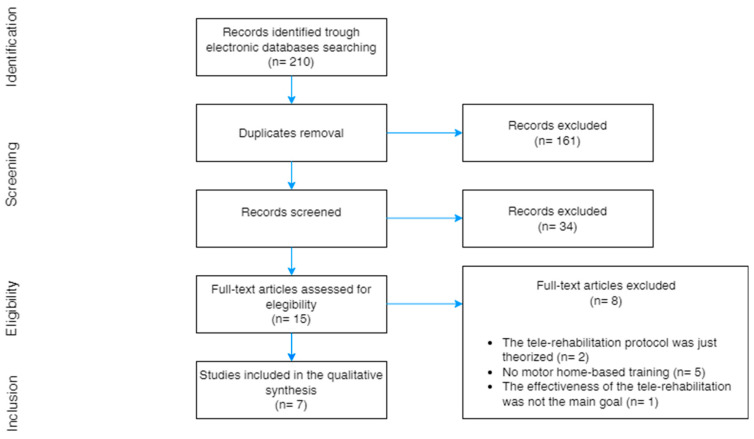
Process of study selection.

A total of 210 articles were retrieved from the previously mentioned electronic databases. After removing the duplicates, the screening of the titles and the abstracts led to the exclusion of 34 items. Out of the remaining 15 articles, 8 failed to meet the inclusion criteria. The selection process and the compete PRISMA flowchart are reported in Figure 1.

Table 1 provides a summary of the articles applying motor tele-rehabilitation interventions to manage the rehabilitation pathway of paucisymptomatic acute and post-acute COVID-19 patients, together with the demographic and clinical characteristics of the evaluated cohorts; details regarding the administration modalities of the tele-rehabilitation programs in terms of tools, duration and specific interventions; and the functional tests performed to assess the patients. To facilitate the comparison of the final results, the baseline of a two-phase study [64] was set at the beginning of the second phase, and it is reported accordingly. Regarding the study cohorts, only data for the patients who completed the rehabilitation path were reported.

The selected studies were analyzed in depth to identify methodological similarities and differences. Specifically, the studies were compared in terms of objectives and participants, experimental protocols and methods for the home-based interventions, functional assessment, and outcomes.

### 3.1. Study Objectives, Participants, and Selection Criteria

The common aim of the selected studies was to assess the effects of a tele-rehabilitation program in order to explore its feasibility and effectiveness in the improvement of physical capacity, quality of life, and management of the symptoms in adults who experioenced COVID-19 infection.

The studies targeted cohorts of patients at different stage of the disease (i.e., paucisymptomatic acute and post-acute) characterized by different magnitudes of symptoms (i.e., from mild to long COVID symptoms).

The studies proposed by Dalbosco-Salas et al. [30], Estebanez-Perez et al. [11], Kortianu et al. [64], Li et al. [22], and Pehlivan et al. [26] targeted adult patients who survived a severe form of COVID-19 and experienced a sequalae of ongoing symptoms for months after the infection. While the study proposed by Estebanez-Perez et al. [11] targeted patients diagnosed with long COVID, regardless of a previous history of hospitalization for inpatient COVID-19 treatments, the other studies addressed previously hospitalized patients. Among them, one study [30] also included non-hospitalized post-COVID individuals, performing a separate analysis of the cohorts based on the patient’s previous status. Beyond details concerning previous hospitalization and/or ICU admittance and patient eligibility according to the defined inclusion criteria, no information was reported on previous treatments or rehabilitation to manage COVID-19 symptoms. Regarding the sociodemographic characteristics, the studies on post-acute patients targeted those with a mean age higher than 45–50 years.

A commonality among the aforementioned studies was the decision to include only patients with moderate dyspnea. Dyspnea was assessed with the modified Medical Research Council (mMRC) scale [22,26,30], a scale [66] normally used to assess the degree of baseline functional disability in patients with respiratory diseases and whose score (ranging from 0 to 4) is associated with the patient’s perception or respiratory symptom burden [67]. According to the baseline evaluation for patient eligibility, post-COVID individuals with mMRC dyspnea higher than 2 were excluded from the programs for safety reasons.

It should also be noted that due to the pandemic situation, it was not always possible to arrange a face-to-face baseline visit to assess the patients [22]. In these cases, patients were evaluated by clinicians via synchronized live video conferences [26].

While most of the analyzed studies targeted post-acute patients, the tele-rehabilitation programs proposed by Rodriguez-Blanco et al. [8,65] addressed patients with ongoing COVID-19 infection reporting mild to moderate symptoms. Patients were recruited through social media, radio programs, and newspapers, and their diagnosis was confirmed by the local epidemiology services during home isolation. Concerning the sociodemographic characterization of the participants, Rodriguez-Blanco et al. [8,65] targeted significantly younger patients (mean age: 37.1 years) than did the studies on post-acute patients.

In addition to inclusion criteria specifically related to the severity and the stage of the disease, it should not be forgotten that tele-rehabilitation methods require a basic knowledge and ability regarding the use of technology [68]. For this reason, only people with sufficient skills related to the use of technological tools (e.g., knowledge concerning accessing email or using a smartphone) were recruited. Moreover, individuals with severe comorbidities (e.g., chronic neurological disorders, chronic kidney disease) preventing the implementation and the safety of the tele-rehabilitation interventions were excluded.

Of the selected studies, only those proposed by Rodriguez-Blanco et al. [8,65], Pehlivan et al. [26], and Li et al. [22] included a control group (CG) that did not participate in tele-rehabilitation.

### 3.2. Experimental Protocols and Methods for Home-Based Interventions

Tele-rehabilitation was performed, addressing different aspects of the rehabilitation needs of COVID-19 patients, using different tools and including different interventions with variable duration.

#### 3.2.1. Target of Tele-Rehabilitation Interventions

Tele-rehabilitation allows for the delivery of multi-disciplinary interventions addressing different aspects and needs [38,69]. As stated in the previous sections, the purpose of this work is to review the state of the art of motor tele-rehabilitation in the management of COVID-19 patients. For this reason, studies which did not specifically targeted motor components [70,71,72] were not included. However, it should be noted that due to the need to treat and address different symptoms, most of the selected studies chose not to administer a monodisciplinary motor rehabilitation program, and they also included simple breathing exercises [22,26,30,64,65], whose role was not explored in depth in this work.

#### 3.2.2. Administration Modality and Tools

Tele-rehabilitation programs were administered either in a synchronous or asynchronous mode, ranging in duration from a minimum of 7 days [8,65] to a maximum of 9 weeks [30]. To execute the interventions correctly, patients were taught to perform the assigned exercises, using small objects (e.g., bands), the inherent technology for tele-rehabilitation (when present), and communication.

In the study conducted by Dalbosco-Salas et al. [30], patients underwent a nine-week asynchronous program consisting in up to 3 weekly home-based tele-rehabilitation session until 24 sessions were reached. Each session lasted up to 45 min and was composed of a short warm up (5 min), simple breathing exercises (3 min), aerobic and/or stretching exercises (up to 30 min), and stretching (5 min). Weekly phone calls were initiated to evaluate user tracking. In this study, the only required device was a phone to communicate with the health center. Conversely, other studies chose to provide the tele-rehabilitation program through a smartphone application designed for home exercise, through which health professionals could define patient-oriented exercise programs [11,22,30,64].

The program proposed by Estebanez-Perez et al. [11] included 3 to 5 weekly sessions for 4 weeks. After a few synchronous sessions via video conference, each training session was performed individually by the patients. Each patient was given recommendations (e.g., walking, jogging) and progressive strength exercises were performed (e.g., glute bridge, spine curl), targeting up to 3 muscle groups with 2 min training intervals via a smartphone app.

A similar modality was also chosen by Kortianu et al. [64], who administered 5 weekly sessions for 8 weeks. The program was delivered in an asynchronous mode, with only 3 sessions supervised by a physiotherapist, and its structure was similar to that proposed by Dalbosco-Salas et al. [30], although with a different duration of the exercise sections. As for the study carried out by Li et al. [22], the 6-week program consisted of 3–4 sessions per week that included breathing control and thoracic expansion, aerobic exercise, and lower limb muscle strength (LMS) exercises. The authors also used a smartphone application, including a chest-worn telemetry device to monitor the heart rate (HR) during the training.

While the studies analyzed so far applied an asynchronous tele-rehabilitation mode, Pehlivan et al. [26] chose a synchronous video conference mode with a physiotherapist, without using a smartphone application to sort the training exercises. The program was delivered in 3 daily sessions for 6 weeks, and it included paced running/self-walking in the corridor, breathing exercises, active cycle of breathing techniques, range of motion exercises, and standing squats.

Regarding the studies proposed by Rodriguez-Blanco et al. [8,65] on paucisymptomatic acute patients, a tele-rehabilitation program including a daily session of 10–30 min per day for a duration of one [8] or two weeks [65] was proposed. The first program consisted of 10 non-specific resistance and strength toning exercises, while in the second study, two separate programs addressing motor or respiratory symptoms were included. As for the motor program, the authors included 10 strength exercises to improve the physical deconditioning and physiological deterioration. In both cases, the programs were carried out asynchronously, although with daily text messages and videoconferences, if required.

In all studies, the number of repetitions of each exercise was adjusted according to the patient’s condition, which was assessed using the Borg Scale [8,26,30,64,65].

### 3.3. Functional Assessment and Outcomes

A functional exercise capacity assessment of the cohorts at pre- and post-treatment was carried out using various functional tests targeting motor performance and muscle strength. Either a single test or a battery of tests was performed at the baseline and after the treatment in order to detect changes in the patient’s functional capacity.

Along with the functional tests, most of the reported studies assessed dyspnea perception after the execution of the tests [8,26,30,64,65], using Borg Scale [8,64,65] or the Modified Medical Research Council dyspnea score [26].

Altogether, the results of the functional tests revealed the effectiveness of the proposed approaches for COVID-19 patients in improving physical capacity, symptomatic status, and consequently, their quality of life. In particular, the outcome of the functional test was characterized by an improving trend between the baseline assessment and the end of the tele-rehabilitation program. Apart from the outcome of such tests, no other measures of physical functioning or disability indexes were provided.

In the studies by Li et al. [22] and Rodriguez-Blanco et al. [8,65], the first test applied for patient assessment was the 6 min walking test (6MWT). The 6MWT is a performance-based measure that was first applied to test exercise tolerance in patients with chronic respiratory disease [73], making it suitable for the assessment of post-COVID patients without severe dyspnea [74]. According to the European Respiratory Society and the American Thoracic society guidelines [75], the test measures the distance walked by an individual over a six-minute period on a flat surface, such as a hospital corridor. To perform the test correctly, the patient is instructed to walk as fast as possible at a self-pace. Rest is also allowed as needed.

In a clinical setting, the test is normally performed in a flat 30 m straight corridor with a mark every 3 or 5 m, with marked turnaround points [22,76,77]. In the reported studies, only Li et al. [22] were able to perform the test in a clinical setting, although only at the follow-up evaluation. In the other studies [8,65], the 6MWT was performed at home and recorded via the patient’s own smartphone. In this case, the patient was asked to walk as far as possible without 180° changes of direction, in order to conform to the variability in the size and distribution of the patient’s house. With respect to the 6MWT, the walked distance after the treatment increased by about 80 m [22] with respect to the baseline.

Dalbosco-Salas et al. [30], Estebanez-Perez et al. [11], and Kortianu et al. [64] assessed their patients with the 1 min sit-to-stand (1 min STS) test [78]. The 1 min STS was proposed as an alternative to the 6MWT, and it represents a reliable tool to measure functional exercise capacity and peripheral muscle performance of the lower limbs in patients with respiratory diseases and airway impairments [11,79,80]. To perform the test, patients were asked to stand up and sit down on a chair for one minute without using the arms or other support. The studies by Rodriguez-Blanco et al. [8,65] proposed a shorter version of the 1-min STS with halved duration, known as the thirty second sit-to-stand test [81].

With respect to the 1 min STS, the number of repetitions increased significantly after tele-rehabilitation, ranging from a minimum of 3 [30] to a maximum of 9–10 [11]. Such results are in line with those of other studies not included in this review [82] and with studies on patients with other diseases [83].

Along with the STS test, Estebanez-Perez et al. [11] and Kortianu et al. [64] evaluated the patients using the short performance physical battery (SPPB), a battery of tests with high internal consistency, which has previously been applied with COVID-19 patients [11,29]. The test consists of a series of timed sub-tests assessing different functional aspects. In particular, gait speed was assessed with the 4 m self-paced walk test, lower extremity strength and endurance with the 5 repetition sit-to-stand (5STS) test, and balance with the standing still with feet together test. Each item has a score ranging from 0 to 4, with a maximum total score of 12.

Pehlivan et al. [26] also applied the SPPB, but combined with the timed up go (TUG) test, a simple test in which the subject is asked to rise from a chair, walk for 3 m, return to the chair, and sit down again. In this case, the score is the time in seconds required to complete the test [84]. Concerning the outcome of the SPBB, Estebanez-Perez et al. [11] and Kortianu et al. [64] increased its parameters with respect to the baseline, indicating an improvement in all the observed functional abilities [11]. In contrast, despite positive developments in regards to the TUG test, Pehlivan et al. [26] did not observe significant differences in the SPPB score.

The 3 min step test (3MST) [64] and the static squat test [22] were also applied to evaluate the patients. In the first case, the patients, starting from a standing position, were asked to step up and down a stair tread in sync with the rhythm provided by a metronome for 3 min straight [85], while in the second case, they were asked to perform a squat against the wall approximating a 90° angle at the hips and knees [86]. In the first case, the recorded parameters were heart rate and oxygen saturation, while in the second case, the score was given by the time in seconds for which patients could maintain the squatting position.

## 4. Discussion

The aim of this review was to provide an overview of the state of the art regarding the application of remote motor rehabilitation programs in the care of paucisymptomatic acute and post-acute COVID-19 patients and an evaluation of their effectiveness in the recovery of functional capacity.

Despite heterogeneity in the duration and intervention protocols, the motor tele-rehabilitation programs reviewed seem to be effective in improving functional exercise capacity and long-lasting symptoms.

The care of post-COVID-19 survivors is a recent challenge for healthcare systems, and there is still no consensus in the literature regarding the therapeutic interventions and functional tests to be used. Although reference values for COVID-19 patients are not available, adopting widely used functional tests allowed for a comparison of the data in the literature. In particular, the 1 min STST has already proven to be an excellent follow-up measure in both face-to-face and remote rehabilitation programs [11,30,64,87]. Although the absence of a control group limits the power of a study [30], the applied functional test contains reference values for asymptomatic subjects.

The comparison of the results achieved by the patients who followed the tele-rehabilitation program with those in the control group showed a difference in the recovery of functional capacity. The difference found in the outcomes of non-hospitalized versus hospitalized patients and non-ICU versus ICU patients reported by Estebanez-Perez et al. [30] is also notable. Although no inter-group differences were found at the baseline, changes in the physical capacity were more significant in the hospitalized group. The latter were hospitalized for one month, adding the effects of prolonged rest [88] to the effects of sedation drugs [89].

Regarding the two studies concerning acute COVID-19 patients with mild-to-moderate symptoms [8,65], the results seem to be in line with authors’ initial hypothesis. In fact, the therapeutic intervention proved to be favorable for acute patients, as it was indicated to strengthen the immune system and improve the respiratory function [90].

The dropout rates, between 10 and 32%, are in line with those reported in the literature for tele-rehabilitation programs for chronic obstructive pulmonary disease (COPD) [30]. Dropout from tele-rehabilitation may occur for various reasons. For instance, it can be due to the loss of human contact, in the case of unsupervised programs, or due to patients’ misperception of their real needs [64]. The dropout rate of the reviewed studies can thus suggest the sustainability of the proposed rehabilitative approaches.

Despite the promising results, the application of the proposed programs requires further exploration. In particular, the limitations could be related to study design and patient assessment, as well as to the use of the technology and to environmental factors.

The main downside related to the study design could be related to the absence of a control group [11,30,64]. In the case of a single-group experimental study, although the results report an improvement in the motor function, as well as in dyspnea and fatigue perception [11], caution should be taken in the interpretation of these data, as it still remains difficult to quantify whether the improvement was due to tele-rehabilitation or to the natural evolution of the disease over time [30,64]. It should also be noted that just one study [22] performed a follow-up evaluation on the patients. Since in the other cases, the evaluation of the patients was performed only at the baseline and the end of the program, it was not possible to investigate what happened in the long term or whether the improvement of the functional capacity was maintained over time.

A risk of bias may be also related to the exclusion of patients with severe dyspnea from tele-rehabilitation. Although this choice was made for safety reasons and to limit the occurrence of adverse events during tele-rehabilitation [22], it seems to limit the generalization of the results in the care of patients with severe dyspnea. In fact, for individuals with severe dyspnea, the same tele-rehabilitation program may not be as effective in the recovery of functional exercise capacity as for pauci- to mild-symptomatic individuals.

In addition, it should also be noted that since no information was provided on the administration of other rehabilitative interventions prior to tele-rehabilitation, it is difficult to determine whether the improvement in functional exercise capacity is due to tele-rehabilitation, to the natural evolution of the disease, or to the long-term effects of a previous treatment. The pandemic caused the suspension of most inpatient and outpatient rehabilitation services, but it is not known whether this suspension affected the rehabilitation path of these patients before tele-rehabilitation and their baseline status. Additionally, due to the restriction of access to healthcare facilities, it was difficult to achieve a complete assessment of the actual functional state of the patients via clinical tests, such as complete spirometry to evaluate pulmonary function [22].

Another bias could be related to the age group of the participants. Only adult patients (>18 years) were assessed, but it seems that only a few studies dealt with patients older than 70 years. For example, in the study of Kortianu et al. [64], the upper age limit was set at 65 years to reduce possible restrictions on technology use and access [91,92]. To ease the difficulty regarding access to technology and to enable a rapid implementation of the tele-rehabilitation process, the authors did not implement any complex platform and chose to use the patients’ own device; this also reduced a possible bias due to subject’s practice of self-assessment due to incorrect use of a new technology. However, this approach limited the generalizability of these findings to the full spectrum of COVID-19 severity and age groups, leading to a selection bias for the patients without access to technology [64]. Conversely, in the studies on paucisymptomatic acute patients by Rodriguez-Blanco et al. [8,65], the sample showed an average age of 40 years. As young individuals are less likely to develop severe symptoms, the actual ability of the studies to assess the preventive role of exercise on hospitalization rate was limited. In addition, the studies by Rodriguez-Blanco et al. [8,65], as pilot examples, were likely underpowered to detect actual changes in physical function, and they did not allow for the assessment of long-term effects, according to the evolution of the symptoms.

As for environmental factors, the main drawback is related to the assessment environment. In fact, among the reported studies, only Li et al. [22] assessed the patients in a clinical setting (although just at follow-up), while in the other studies, patients were assessed via tele-conference in their home setting. Despite the author’s effort to adapt the test setup (e.g., 6 min walking test) to contain the variability of the patient’s homes, it was difficult to rule out experimental bias or to ensure the actual reliability of functional tests in domestic settings [64].

This is a narrative review and therefore, quantitative approaches to assess the quality of the studies commonly used in systematic reviews were not used [93,94]. In addition, it was chosen to limit the review to studies applying motor tele-rehabilitation interventions, thus excluding those focusing on the respiratory aspects [70,71,72,95].

The difference between studies applying only motor interventions and those applying either motor and respiratory interventions was not explored in the present work. Since in the work of Rodriguez-Blanco et al. [65], the study groups that performed a strength-based and a breathing-based rehabilitation program both showed clinical improvements in patients’ conditions, it would be interesting to examine works combining both approaches for tele-rehabilitation.

Several case series and studies have been proposed, aiming to improve the respiratory system in COVID-19 patients [70,71,72]. For example, in the study proposed by del Corral et al. [70], the authors presented a respiratory tele-rehabilitation program for post-COVID patients, specifically targeting both inspiratory and expiratory muscles. The results of this study reported that inspiratory and respiratory muscle training improved breathing muscle function, as well as lower limb muscle strength, according to the results of the 1 m STST.

Given the benefits emerging from these studies at both motor and respiratory levels, as well as the close link between exercise capacity and respiratory function reported in literature [96,97], in the future, it may be possible to expand this review to include studies applying respiratory-oriented tele-rehabilitation programs.

## 5. Conclusions

The mechanisms underlying the functional recovery of post-COVID-19 patients are not yet fully elucidated, and the individual differences in terms of ongoing symptoms makes it difficult to define standardized rehabilitation protocols. Currently, tele-rehabilitation seems to be a feasible, safe, and effective complementary tool in managing the rehabilitation needs of post-COVID patients, opening new perspectives for a tele-health care-delivery model [98,99], but it does not replace face-to-face interventions [100].

## Figures and Tables

**Table 1:** Summary of the main details of the reviewed studies. TeleG = group performing tele-rehabilitation; CG = control group.

				Tele-Rehabilitation Program			
Source	Year and Country	Study Type	#Participants, Age (Years), Gender (#M/F) and Stage of the Disease	Type	Mode, Tools, and Duration	Interventions	Functional Assessment of Physical Capacity
Dalbosco-Salas et al. [30]	2021 Chile	Multicentric, observational, and prospective study	Total = 115 (M: 46/F: 66) Age = 55.6 ± 12.7 years Hospitalized (n = 57) and Non-Hospitalized (n = 58) Post-COVID patients	Motor and Respiratory	2–3 d/w for 9 weeks (24 sessions) Weekly phone calls	Warm up (5 min), breathing exercises (3 min), aerobic and/or strength exercises (20–30 min), and stretching (5 min)	1 min sit-to-stand test (1 min STST)
Estebanez-Pérez et al. [11]	2022 Spain	One arm quasi-experimental clinical trial	Total = 32 (M: 9/F: 23) Age = 45.93 ± 10.65 years Long-COVID patients	Motor	3–5 d/w for 4 weeks Synchronous sessions via video conference and sessions via smartphone app	Personalized recommendations (e.g., walking, jogging) and progressive strength training, working 1–3 muscle groups with a load of 8–12 reps, with 2 min training intervals	1 min STST Short performance physical battery test (SPPB)
Kortianou et al. [64]	2022 Greece	Single-cohort interventional study	Total = 22 (M: 18/F: 4) Age = 50.1 ± 13.2 years Hospitalized Post-COVID Patients	Motor and respiratory	5 d/w for 8 weeks Daily unsupervised self-practice via smartphone app 3 supervised sessions with a physiotherapist over the duration of the program	Warm-up (5–10 min), aerobic and total body strengthening exercises (15–20 min), and stretching (5–10 min)	1 min STST SPPB 3 min step test (3MST)
Pehlivan et al. [26]	2022 Turkey	Randomized controlled study	Total = 34 TeleG = 17 (M:14/F:3) Age = 50.76 years CG = 17 (M:11/F:6) Age = 43.24 years Post-COVID patients	Motor and respiratory	3 d/w for 6 weeks Synchronous video conference with a physiotherapist	Paced running/self-walking in the corridor, breathing exercises, active cycle of breathing technique, range of motion exercises, and standing squat	Timed up and go test (TUG) Short physical performance battery (SPPB)
Li et al. [22]	2022 China	Parallel-group randomized controlled trial	Total = 119 TeleG = 59 (M:27/F: 32) Age = 49.17 ± 10.75 years CG = 60 (M: 26 F:43) Age = 52.03 ± 11.10 years Post-COVID patients (formerly hospitalized)	Motor and respiratory	3–4 d/w for 6 weeks Daily self-practice via smartphone app	Breathing control and thoracic expansion, aerobic exercise, and LMS exercise	6 min walking test (6MWT) Squat test
Rodriguez-Blanco et al. [8]	2021 Spain	Randomized controlled trial	Total = 36 TeleG = 18 (M: 9/F: 9) Age = 39.39 ± 11.74 years CG = 18 (M:8/F:10) Age = 41.33 ± 12.13 years Acute patients with mild to moderate symptomatology	Motor	Daily 10–30 min session for one week Daily text messages and videoconferences, if needed	10 non-specific toning exercises of resistance and strength up to 12 reps	6 min walking test (6MWT) Thirty second sit-to-stand test (30STST)
Rodriguez-Blanco et al. [65]	2022 Spain		Total = 77 TeleG (1) = 26 (M: 14/F: 12) Age = 34.81 ± 11.82 years TeleG (2) = 29 (M:13/F: 16) Age = 41.93 ± 10.19 years CG = 22 (M: 13/F: 12) Age = 42.36 ± 11.84 years Acute patients, with mild to moderate symptomatology	Motor or respiratory	Daily 10–30 min session for 14 days Daily text messages and videoconferences, if needed	TeleG (1): 10 strength exercises to improve the physical deconditioning and physiological deterioration TeleG (2): 10 exercises based on the active cycle of breathing techniques	6MWT 30STST

## Data Availability

Not applicable.
